# Metabolomic changes in the mouse retina after optic nerve injury

**DOI:** 10.1038/s41598-018-30464-z

**Published:** 2018-08-09

**Authors:** Kota Sato, Daisuke Saigusa, Ritsumi Saito, Amane Fujioka, Yurika Nakagawa, Koji M Nishiguchi, Taiki Kokubun, Ikuko N. Motoike, Kazuichi Maruyama, Kazuko Omodaka, Yukihiro Shiga, Akira Uruno, Seizo Koshiba, Masayuki Yamamoto, Toru Nakazawa

**Affiliations:** 1 Department of Ophthalmology, Tohoku University Graduate School of Medicine, Sendai, Miyagi Japan; 2 Department of Ophthalmic imaging and information analytics, Tohoku University Graduate School of Medicine, Sendai, Miyagi Japan; 3 Department of Integrative Genomics, Tohoku Medical Megabank Organization, Tohoku University, Sendai, Miyagi Japan; 4 Medical Biochemistry, Tohoku University School of Medicine, Sendai, Miyagi Japan; 5 LEAP, Japan Agency for Medical Research and Development (AMED), Chiyoda, Tokyo Japan; 6 Department of Advanced Ophthalmic Medicine, Tohoku University Graduate School of Medicine, Sendai, Miyagi Japan; 7 Department of Systems Bioinformatics, Graduate School of Information Sciences, Tohoku University, Sendai, Miyagi Japan; 8 Department of Innovative Visual Science, Graduate School of Medicine, Osaka University, Suita, Osaka, Japan; 9 Department of Retinal Disease Control, Tohoku University Graduate School of Medicine, Sendai, Miyagi Japan

## Abstract

In glaucoma, although axonal injury drives retinal ganglion cell (RGC) death, little is known about the underlying pathomechanisms. To provide new mechanistic insights and identify new biomarkers, we combined latest non-targeting metabolomics analyses to profile altered metabolites in the mouse whole retina 2, 4, and 7 days after optic nerve crush (NC). Ultra-high-performance liquid chromatography quadrupole time-of-flight mass spectrometry and liquid chromatography Fourier transform mass spectrometry covering wide spectrum of metabolites in combination highlighted 30 metabolites that changed its concentration after NC. The analysis displayed similar changes for purine nucleotide and glutathione as reported previously in another animal model of axonal injury and detected multiple metabolites that increased after the injury. After studying the specificity of the identified metabolites to RGCs in histological sections using imaging mass spectrometry, two metabolites, i.e., L-acetylcarnitine and phosphatidylcholine were increased not only preceding the peak of RGC death in the whole retina but also at the RGC layer (2.3-fold and 1.2-fold, respectively). These phospholipids propose novel mechanisms of RGC death and may serve as early biomarkers of axonal injury. The combinatory metabolomics analyses promise to illuminate pathomechanisms, reveal biomarkers, and allow the discovery of new therapeutic targets of glaucoma.

## Introduction

Glaucoma is neurodegenerative disease that leads to irreversible visual field defects. It is characterized by a chronic and progressive loss of retinal ganglion cells (RGCs)^1–3^. Previous research has shown that many mechanisms contribute to RGC death, including calpain activation, glutamate excitotoxicity, oxidative stress and ER stress^4–7^. Recently, transcriptomic, proteomic and metabolomic approaches have been used to identify pathological pathways in glaucoma^8–10^. However, the results of these studies might not reflect events specific to dying RGCs, because they were based on whole retinal samples, even though the RGCs are only present in the ganglion cell layer (GCL) of the retina. One method of overcoming these limitations is to study RGC death with mass spectrometry (MS) imaging, which can detect metabolites in specific regions of tissue sections.

Recently, metabolic profiling has been proposed as a technique for identifying new biomarkers. This technique, which has been enabled by improvements in MS-based technologies, can detect new metabolites and reveal new candidate biomarkers. In the retinal research field, the effect of optic nerve crush (NC) on amino acids, carbohydrates, lipids, and the metabolism of energy (glucose and the tricarboxylic acid cycle) has been studied, and MS-based methods have been found to be able to clearly discriminate control subjects from those with early or late injuries^10^. Previous research based on a targeted metabolomic analysis revealed that oxidative stress, energy depletion and the ceramide pathway were involved in retinal damage in rats after NC^10^. However, targeted metabolomics has a limited capacity to identify new biomarkers. By contrast, global metabolomics (G-Met) technologies, such as untargeted metabolic profiling with liquid chromatography-MS (LC-MS), have a high potential to reveal new biomarkers. Recently, retinal biomarkers of hypoxia have been identified with ultra-high-performance LC quadrupole time-of-flight MS (UHPLC-QTOF/MS) and UHPLC triple quadrupole MS (UHPLC-MS/MS)^11^. However, it will be necessary to assess a greater number of metabolites, including metabolites with a wide range of polarities, to find biomarkers of disease progress. To this end, UHPLC-QTOF/MS equipped with a reverse-phase (C18) column was previously combined with LC Fourier transform mass spectrometry (LC-FTMS) equipped with a normal-phase (hydrophilic interaction chromatography, HILIC) column to establish a method of performing G-Met for a wide range of metabolites^12^.

In this study, we show that global metabolomic analysis by combinatory methods revealed metabolites that may be linked to novel disease mechanisms of axonal injury. Furthermore, temporal profiling and histological analysis of the metabolites revealed that some are altered in the RGCs preceding their death. These findings may lead to the development of early biomarker of RGC death and aid the development of new therapeutic strategies for glaucoma.

## Results

### Retinal ganglion cells were injured over time after optic nerve injury in mice

**Figure 1:**
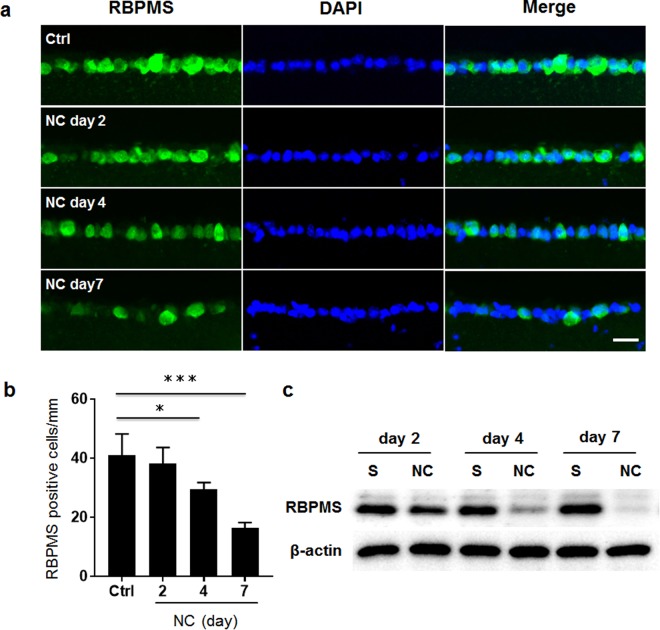
RGC degeneration after NC in the mouse retinas. (**a**) Immunohistochemistry of RBPMS (green), an RGC marker, after staining in the retinas of uninjured controls (Ctrl) and 2, 4 or 7 days after NC. Nuclei were counterstained with DAPI (blue). The scale bar is 20 μm. (**b**) Histogram showing the average number of RBPMS-positive cells in the GCL. The error bars designate SD (N = 4). ^*^P < 0.05, ***P < 0.001. (**c**) The protein level of RBPMS in the retina was measured with an immunoblot analysis. Beta actin was used as an internal control. S: sham operation in contralateral eyes of the NC eyes.

To confirm that the RGCs were damaged after NC, we assessed the expression level of RBPMS as a marker of the RGCs^13^. Quantitative measurements showed that the number of RBPMS-positive cells in the GCL of the mouse retinas was slightly reduced 2 days after NC, compared with uninjured control retinas (uninjured retinas: 40.9 ± 7.3 cells/mm; 2 days after NC: 38.3 ± 5.3 cells/mm). The number of RBPMS-positive cells decreased significantly 4 days after NC (29.4 ± 2.3 cells/mm) and 7 days after NC (16.3 ± 1.9 cells/mm) (Fig. 1a,b). Western blotting analysis also showed that the level of RBPMS protein gradually decreased after NC in the mouse retinas (Fig. 1c), confirming that degeneration of the RGCs had occurred after NC in the mouse retinas.

### Principal component analysis and discriminant analysis

**Figure 2:**
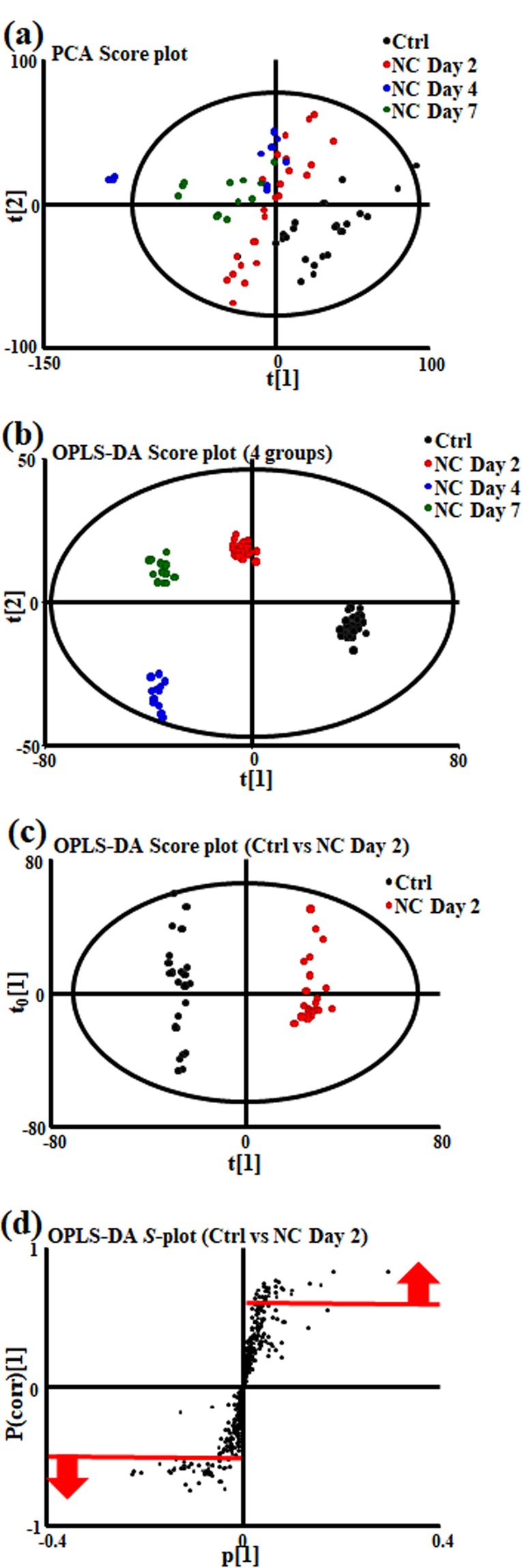
(**a**) PCA score plot visualizing changes in the metabolomic profile of the mouse retina. The black, red, blue and green symbols indicate control (Ctrl), nerve crush (NC) day 2, NC day 4 and NC day 7 sample groups, respectively. (**b**) OPLS-DA score plot visualizing changes in the metabolomic profile of the retina in the above 4 groups. (**c**) OPLS-DA score plot visualizing changes in the metabolomic profile of the mouse retina in the Ctrl and NC day 2 groups. (**d**) S-plot analysis of OPLS-DA data to extract features profiles of the retina in the Ctrl and NC day 2 groups. Features higher in the NC day 2 group are described by the following area: 0 < p[1], p(corr)[1] < 1. Features lower in the NC day 2 group are described by the following area: −1 < p[1], p(corr)[1] < 0. The red lines indicate correlation values (p(corr)[1]P) greater than 0.6 (up to 1.0) and less than −0.55 (as low as −1.0) for the selected features.

After *t*_R_ alignment and deconvolution with Progenesis QI, the HILIC column in positive ion (HILICpos) mode revealed 5,902 features, the negative ion (HILICneg) mode revealed 1,833 features, the C18pos mode revealed 7,675 features, and the C18neg mode revealed 5,398 features. We then applied a normalization procedure for the abundance of retinal metabolites. After normalization, there were 1,578 HILICpos assay-detected features, 272 HILICneg-detected features, 1,194 C18pos-detected features, and 1,645 C18neg-detected features. All these features passed our selection criteria for a multivariate analysis of the retinal samples, which we obtained from control (Ctrl) animals and on days 2, 4, and 7 after NC. Figure 2a,b shows a typical principal component analysis (PCA) score plot and an orthogonal partial least square-discriminant analysis (OPLS-DA) score plot from the C18pos analysis for all four groups of mice. These results clearly show large variations between the retinas from the Ctrl and NC groups. Next, based on our OPLS-DA findings, we selected features that contributed to the differences between the Ctrl and NC day 2 groups. We defined large changes in abundance based on a correlation value (p(corr)[1]P) greater than 0.60 (up to 1.0) and less than −0.55 (as low as −1.0), as derived from *S*-plot analyses in the four assays (Fig. 2c,d). All selected features in the four assays are listed in Supplementary Table 1.

### Metabolomic Analysis

**Figure 3:**
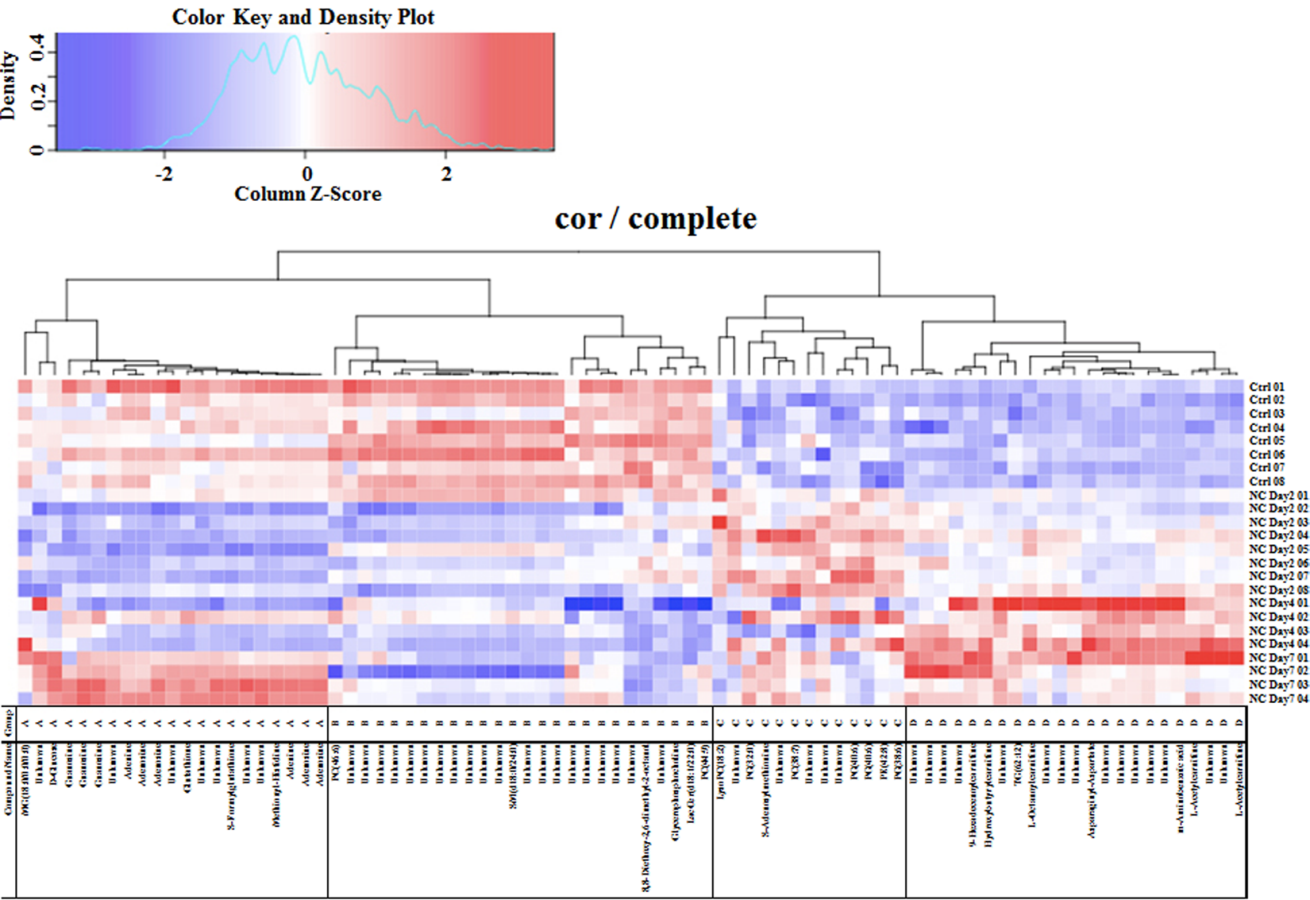
Hierarchical cluster analysis of retinal metabolites in control animals and in mice on days 2, 4 and 7 after optic nerve crush (cor/complete).

Eighty-three metabolites in the mouse retinas had significantly different concentrations after NC when compared with the non-treated group, (P < 0.01) and 30 of these 83 metabolites were identifiable by UHPLC-QTOF/MS and LC-FTMS (Table 1). To analyze these metabolites in further detail, we sorted them with hierarchical clustering and classified them into 4 subgroups (Fig. 3).

**Table 1:** The list of metabolites identified by the S-plot in four analytical modes: C18 column positive ion mode, C18 column negative ion mode, HILIC column positive ion mode and HILIC column negative ion mode of the G-Met. The metabolites were divided into four groups (A, B, C and D) with a hierarchical cluster analysis.

Group	Mode	m/z	rt	ID	Name
A	HN	357.30206	0.72	HMDB11131	MG(18:0/0:0/0:0)
A	CN	225.06047	0.81		Unknown
A	HN	179.0565	5.32	HMDB00122	D-Glucose
A	CN	282.08356	3.98	HMDB00133	Guanosine
A	HN	282.08495	4.28	HMDB00133	Guanosine
A	HP	284.09893	4.28	HMDB00133	Guanosine
A	HN	332.06219	3.39		Unknown
A	CP	136.06228	4.35	HMDB00034	Adenine
A	CP	268.10437	4.35	HMDB00050	Adenosine
A	CN	312.0942	4.35	HMDB00050	Adenosine
A	HN	329.08597	3.39		Unknown
A	HN	288.07207	3.37	HMDB00125	Glutathione
A	HN	302.06675	3.39	HMDB29395	Unknown
A	HN	477.17838	3.39		Unknown
A	HN	334.07777	3.39	HMDB01550	S-Formylglutathione
A	HN	350.07282	3.39		Unknown
A	HN	533.18688	3.39		Unknown
A	HN	267.09321	3.39	HMDB28975	Methionyl-Histidine
A	HN	134.04745	3.39	HMDB00034	Adenine
A	HN	266.08988	3.39	HMDB00050	Adenosine
A	HN	312.09566	3.39	HMDB00905	Adenosine
B	CP	918.70672	0.72	HMDB08781	PC(46:6)
B	CP	203.05303	0.81		Unknown
B	CP	326.88686	0.7		Unknown
B	CP	546.82285	0.71		Unknown
B	CP	614.80996	0.73		Unknown
B	CP	394.87417	0.7		Unknown
B	CP	598.83628	0.71		Unknown
B	CP	226.95182	0.7		Unknown
B	CP	378.90019	0.7		Unknown
B	CP	446.88761	0.7		Unknown
B	CP	530.84841	0.72		Unknown
B	CP	462.86163	0.7		Unknown
B	CP	834.74528	0.72	HMDB12094	SM(d18:0/24:0)
B	CP	902.7309	0.67		Unknown
B	CP	682.79743	0.71		Unknown
B	CP	750.78378	0.71		Unknown
B	CP	253.03315	0.82		Unknown
B	CP	398.03236	0.82		Unknown
B	CP	501.066	0.82		Unknown
B	CP	751.09497	0.82		Unknown
B	CP	217.10496	4.63		Unknown
B	CP	269.20908	10.43	HMDB34557	8,8-Diethoxy-2,6-dimethyl-2-octanol
B	CP	887.10094	0.82		Unknown
B	CP	258.11078	0.85	HMDB00086	Glycerophosphocholine
B	HP	946.72552	0.95	LMSP0501AB06	LacCer(d18:1/22:0)
B	HP	906.59931	0.95	HMDB08648	PC(44:9)
C	CP	520.33884	9.83	HMDB10386	LysoPC(18:2)
C	HP	244.22638	0.88		Unknown
C	HP	740.54344	0.58	HMDB10570	PG(32:0)
C	HP	399.14445	5.4	HMDB01185	S-Adenosylmethionine
C	HN	327.05779	3.89		Unknown
C	HP	804.55391	0.96		PC(38:7)
C	HP	195.76274	1.09		Unknown
C	HP	284.25829	0.75		Unknown
C	HP	815.55978	0.58	HMDB10604	Unknown
C	HP	840.57405	0.57	HMDB10614	PG(40:6)
C	HN	821.53613	0.54	LMGP04010040	PG(40:6)
C	HN	796.52306	0.55		PE(42:8)
C	HN	793.50564	0.54	HMDB10584	PG(38:6)
D	CP	292.84554	0.73		Unknown
D	CN	516.81731	0.74		Unknown
D	CN	364.86896	0.74		Unknown
D	HP	246.13287	3.83		Unknown
D	HP	398.32634	2.87	HMDB13207	9-Hexadecenoylcarnitine
D	HP	248.14848	4.2	HMDB13127	Hydroxybutyrylcarnitine
D	HP	332.09639	3.86		Unknown
D	HP	996.8006	0.66		TG(62:12)
D	HP	288.21833	3.1	HMDB00791	L-Octanoylcarnitine
D	HP	117.06524	5.04		Unknown
D	HP	160.09504	4.41		Unknown
D	HP	262.16492	4.02		Unknown
D	HN	228.06473	4.98	HMDB28727	Asparaginyl-Aspartate
D	CP	367.13975	0.9		Unknown
D	HN	182.05914	4.98		Unknown
D	HN	244.05957	4.98		Unknown
D	HP	120.10123	5.05		Unknown
D	HP	139.05725	4.09		Unknown
D	HP	138.05392	4.09	HMDB01891	m-Aminobenzoic acid
D	CP	204.12349	0.98		L-Acetylcarnitine
D	HN	348.09228	4.04		Unknown
D	HP	204.04345	4.06	HMDB00201	Unknown
D	HP	204.12053	4.04	HMDB00201	L-Acetylcarnitine

Group A contained 9 metabolites that decreased on days 2 and 4 after NC, but increased again on day 7. Group B contained 6 metabolites that maintained a consistently reduced level after NC. Group C contained 8 metabolites that transiently increased on day 2 after NC and then decreased slightly on days 4 and 7. Group D contained 7 metabolites that increased gradually and peaked on day 4 after NC. The metabolites that were identified are listed in Table 1.

### Imaging MS Analysis

**Figure 4:**
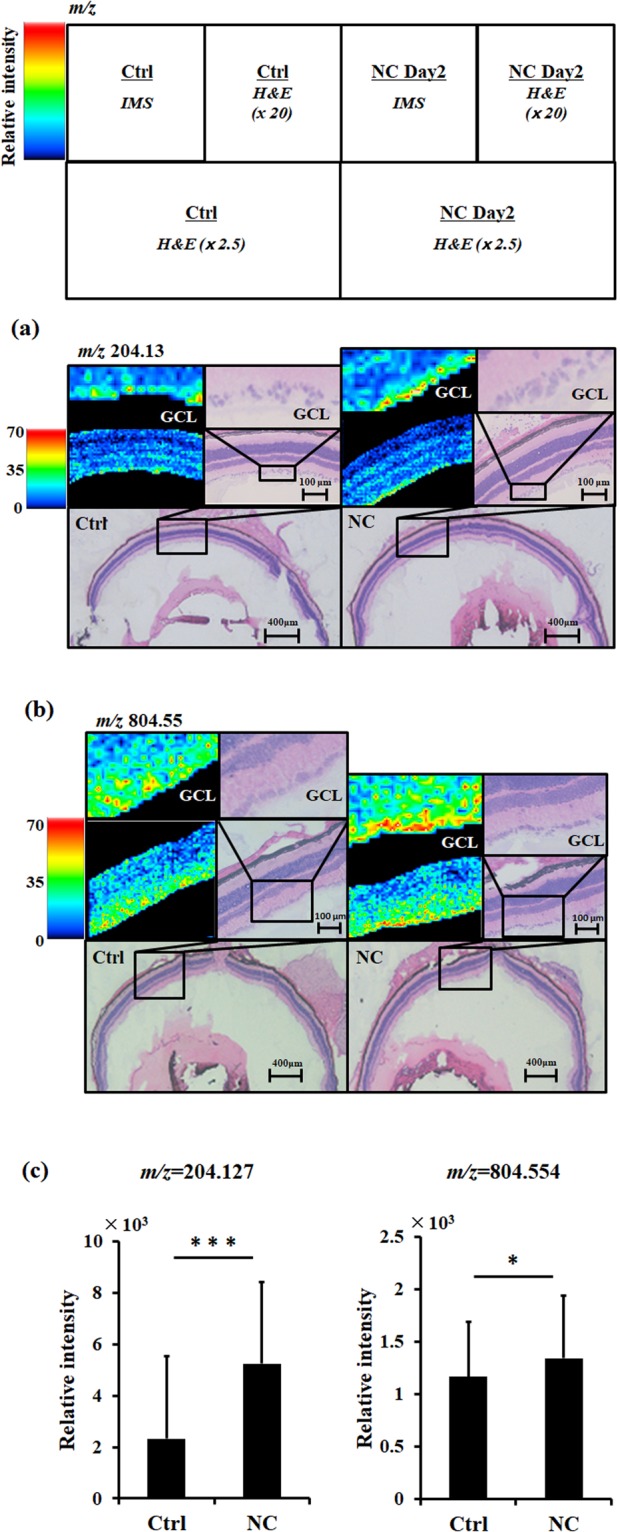
Distribution of representative metabolites (listed in Fig. 2), as evaluated with G-Met assays. The following metabolites had a higher intensity in the ganglion cell layer of the eyes of the mice on NC day 2, as measured with imaging mass spectrometry (IMS) assays: (**a**) m/z 204.13, L-acetylcarnitine and (**b**) m/z 804.55, phosphatidylcholine (38:7). A and B are microscopic images showing hematoxylin-eosin staining in cryosections of the eyes (x 2.5), with the retinal area around the optic disk also shown at higher magnification (x 20). These images were obtained after IMS. Relative intensities are shown by the colored scale bar on the left side of the IMS data. (**c**) Histograms showing the relative intensity at each point, calculated and denoted with the standard deviation. ^*^P < 0.05, ^***^P < 0.001.

Next, we used IMS to find the locations in the retina of the identified post-NC changes in RGC metabolites. Increases in metabolites such as L-acetylcarnitine (*m/z* 204.3) and PC (38:7) (*m/z* 804.55) were detected. These metabolites significantly increased in the GCL in mouse retinal sections (control: 2326.2 ± 1387.4 vs. NC day 2: 5247.1 ± 3197.0 as L-acetylcarnitine, control: 1167.8 ± 525.3 vs. NC day 2: 1345.2 ± 600.7 as PC (38:7), Fig. 4a–c). These changes were consistent with the results of our MS spectrometric analysis (Fig. 3). IMS was also able to detect other metabolites, including adenine, glycerophosphocholine, adenosine, lysophosphatidylcholine (LPC) (18:2) and phosphatidylglycerol (32:0), but could not detect the precise location of changes in these metabolites in the GCL (Supplementary Fig. 1).

## Discussion

This study used G-Met to identify new phospholipids in the retina after NC, in addition to sugar, amino acids and purine nucleobases. We categorized these metabolites into four groups with a hierarchical analysis: among the metabolites in these groups, purine metabolites, D-glucose and glutathione metabolites decreased in the early stages of RGC damage (before significant RGC loss), and then increased in the late stages. Glycerophosphatidylcholine and LacCer decreased continuously after NC. Most elevated metabolites in the early stages of RGC damage were phosphatidylglycerol (PG) metabolites, and carnitine metabolites in the late stages. Moreover, some phospholipids with altered levels in the retina after NC were observed to be specifically elevated in the GCL. These findings demonstrate that our new method, which combines LC-MS and IMS, can identify new, promising biomarkers and should help in the understanding of the pathophysiological mechanisms associated with RGC-specific neuronal degeneration.

Although dysfunctional RGCs can be observed in the retina in the early stages of glaucoma^14^, these cells represent only a small proportion of the total cells in the retina. Therefore, analyses based on the entire mass of the retina are less likely to accurately detect the distribution of metabolites specific to the RGCs, or to provide sufficient sensitivity and spatial resolution to adequately reveal pathophysiological events in the RGCs. A promising alternative is matrix assisted laser desorption/ionization imaging MS (MALDI IMS) technology, which has high enough spatial resolution to allow the distribution analysis of metabolites^15^. Methods for distribution analysis focused on the central metabolites and on lipids in the retina have been established based on MALDI IMS^16–19^. Furthermore, the sensitivity and spatial resolution of focused lipid localization analysis have been found to be high^20^. Therefore, the combination of LC-MS and MALDI IMS technology for G-Met promises to reveal new prognostic biomarkers of glaucoma, and to enable new ways of identifying the disease. Additionally, this method may clarify the underlying pathological mechanisms, and should help to find new ways to prevent disease progression at an early stage.

In this study, metabolites in group A decreased 2 days and 4 days after NC, but increased on day 7, in comparison with the non-treated retinas. Group A included guanosine and adenosine, which are classified as purine nucleosides and are well known to modulate the homeostatic function of Muller cells, astrocytes and microglia^21–23^. In addition, purinergic signaling protects against cell death in injured neurons. For example, guanosine has a neuroprotective effect in the hippocampus, reducing oxidative stress and inhibiting the PI3K/Akt/GSK3b pathway-mediated inflammatory response and iNOS^24–27^. Adenosine inhibits P2X7 receptor-induced calcium influx and protects the RGCs^28,29^. Nicotinamide adenine dinucleotide (NAD), one of the products of adenine, prevents axonal loss and protects against cell body degeneration in the RGCs^30,31^, suggesting that adenine might contribute to the maintenance of the RGCs.

The metabolic cycle of glutathione, which is regulated by glutathione reductase (GSR) and glutathione peroxidase (Gpx1), is well known to include a redox function and to defend cells from oxidative-stress injury^32^. Previous work has shown that the glutathione level in blood samples taken from glaucoma patients is lower than in age-matched controls^33^. Our data also showed that the glutathione level in the retina on day 2 after optic nerve crush was less than in the uninjured retinas. These findings suggest that lower levels of glutathione in glaucoma patients might be associated with optic nerve damage, and that they may cause progressive RGC loss via insufficient redox. The reduction of glutathione after NC may also be due to defective enzymatic regulation.

Interestingly, the metabolites in group A that had decreased in the earlier stages increased on day 7 after NC, reaching levels that were higher than the non-treated baseline. It is known that nucleotides and nucleosides released from injured neurons activate the microglia. Furthermore, adenosine- and ATP-induced microglial activation promotes the recruitment of microglia around the site of neuronal damage, acting via the A3 and P2Y12 receptors^34^. Previous research has also revealed that on day 7 after NC, most RGCs disappear from the retina and macrophages and microglia are recruited^35^. This suggests that in group A, the recruitment of microglia was regulated by adenosine and ATP released from injured RGCs.

Metabolites in group B maintained a consistently reduced level after NC and were mostly classified as chorines. Phosphatidylcholines are one of the components of the plasma membrane and are catalyzed by phospholipase D (PLD)^36^. In damaged RGCs in mice after NC, the plasma membrane may be disrupted by PLD activation. Chorine has crucial roles in maintaining plasma membranes, in the synthesis of neurotransmitters (in which it acts as a substrate) and in supplying methyl groups with material. LacCer (d18:1/22:0), categorized as a sphingolipid, is also a component of plasma membranes. Citicoline, an exogenous CDP-choline, has been shown to improve retinal function in glaucoma patients and to prevent apoptotic cell death in the RGCs^37–40^. Previous studies also suggest that the supply of choline has a crucial role in maintaining the RGCs and visual function, and that low levels of cholines after optic nerve injury may contribute to glaucoma progression.

Metabolites categorized in group C increased gradually and peaked on day 2 after NC, suggesting that metabolites in group C have potential as predictive biomarkers of RGC damage during the early stages. Interestingly, retinal metabolites in group C included elevated lysophosphatidylcholine (LPC) levels. Previous work has demonstrated that exposure to LPC induces demyelination and inflammation of the optic nerve in a model of optic neuritis^41^. LPC also induces the recruitment of macrophages and modulates neutrophil oxidant production^42^. Here, we also found that phosphatidylcholine (PC) (38:7) was elevated in the GCL after NC in mice. The pathophysiological effects of elevated LPC might contribute to RGC degeneration after NC via demyelination, inflammation and oxidant production. In addition, LPC contributes to age-related macular degeneration (AMD), suggesting that LPC might be involved not only in pathological neurodegeneration, such as occurs during glaucoma progression, but also in neovascularization in other retinal diseases.

Another interesting finding of this study was that PG (16:0/16:0) and PG (18:0/22:6), both included in group C, increased after NC. PG is converted from lysophosphatidylglycerol by lysophospholipid acyltransferase (Lpt1p)^43^. Elevated PG in the mouse retina after NC may depend on the activation of Lpt1p and promote the production of cardiolipin, which is a catalysis product of PG. Cardiolipin, a subspecies of PG, is a component of the mitochondrial inner membrane. Cardiolipin is needed for the translocation of caspase-8 to the mitochondria in apoptotic cells^44^. Cardiolipin is also required for the functioning of inflammasome, and directly binds with Nlrp3 inflammasome^45^. In injured cells, PG is released from the mitochondria and induces the activation of apoptosis via cytochrome C, caspase 8 and inflammasome signaling. Autoantibodies against PG have been found in glaucoma patients^46^. This suggests that axonal damage induces excess levels of PG in the RGCs, and that it might be involved in the loss of RGCs in glaucoma via apoptosis signaling, inflammation and autoantibody antigens.

S-adenosylmethionine (SAM), a metabolite of polyamine and an important methyl donor in cells, also increased after NC in the mice in this study, and was included in group C. SAM is synthesized in a reaction catalyzed by methionine adenosyltransferases (MATs)^47^ and catabolized with glycine N-methyltransferase (GNMT)^48^. Previous studies have reported that SAM has a neuroprotective effect against L-dopa toxicity *in vitro* and ischemic damage *in vivo*^49–51^. SAM also functions to reduce inflammation via the suppression of oxidative stress in a mouse model of chronic asthma^52^, and is well known as a substrate of spermidine and spermine. Spermidine treatment promotes autophagy, suppressing oxidative stress and necrosis^53^. In the retina, SAM restores photoreceptor function^54^ and protects the RGCs in a mouse model of glaucoma^35,55^. SAM not only has a neuroprotective effect, but can also promote optic nerve regeneration^35^. These previous findings suggest that the elevation in SAM we observed after optic nerve crush in mice might act to promote RGC survival and regenerate the optic nerve. In the current study, glutathione decreased 2 days and 4 days after NC. SAM plays an important role in the synthesis of glutathione by providing homosysteine. Glutathione reduction and SAM elevation after NC may be due to the inhibition of some enzymatic activity related to SAM and the glutathione pathway, including the activity of enzymes including GNMT, S-adenosylhomocysteine hydrolase (SAHH) and cystathionine beta-synthase (CBS). Additionally, SAM is a methyl donor and converts phosphatidylethanolamine (PE) to phosphatidylcholine (PC)^56^. In the current study, PE (42:8) increased and PC decreased slightly after NC, suggesting that the conversion of SAM to S-adenosylhomocysteine (SAH) by PE N-methyltransferase (PEMT) and GNMT may be suppressed after NC. PE (42:8), was also included in group C in this study. It is associated with Raf-1 kinase inhibitory protein (RKIP), which functions to promote RGC survival and axonal regeneration after NC in mice^57^. It is possible that elevated PE (42:8) is also part of a protective response in injured RGCs after NC.

Metabolites categorized in group D increased gradually and peaked on day 4 after NC, suggesting that metabolites in group D have potential as predictive biomarkers of RGC damage during the advanced stages. All identified metabolites in group D were categorized as carnitines. Additionally, L-acetylcarnitine increased in the GCL after NC. L-acetiylcarnitine is synthesized by carnitine acetyltransferase (CAT) from acetyl-CoA and carnitine^58,59^. The elevation of L-acetylcarnitine after NC may be due to the increase or activity of CAT in the mouse retina. Acetylcarnitine can improve mitochondrial function via attenuation of mitochondrial protein acetylation.^60^ Carnitine treatment has been shown to prevent the loss of RGCs in the retinas of mice with induced high IOP, acting as an antioxidant. It is possible that this is because L-acetylcarnitine is the main component of the inner membrane of mitochondria^61^. Thus, elevated carnitine may be a biomarker of progressive damage in the RGCs, and elevated carnitine may play a neuroprotective role in maintaining mitochondrial function during RGC degeneration after NC in mice. Previously, we found that SYTOX orange-positive dead RGCs were mainly detectible on day 4 after NC^62^. The current study confirms that L-acetylcarnitine was also highly elevated 4 days after NC in the GCL. This suggests that elevated L-acetylcarnitine is associated with RGC death, and that it might be a candidate biomarker of RGC death. However, the GCL contains not only RGCs, but also other cells, such as displaced amacrine cells. Therefore, amacrine cells may also have increased L-acetylcarnitine and PC (38:7) in the GCL after optic nerve crush. However, it is hard to determine which cells upregulate these metabolites in the GCL. This was therefore a limitation of the present study that will require further consideration in a future study.

In conclusion, our MS-based approach identified novel metabolites that changed in the mouse retina after NC. Categorizing the identified metabolites into subgroups provided insights that may lead to the discovery of previously unknown pathomechanisms and offer a better understanding of already identified mechanisms of RGC degeneration after NC. In particular, IMS technology allowed the identification of new potential biomarkers of RGC damage: PC (38:7) in the early stages and L-acetylcarnitine in the advanced stages. These metabolomic alterations, including alterations in phospholipids, may help the clinical diagnosis of glaucoma and lead to the discovery of new candidate molecules for therapies targeting RGC loss caused by axonal injury.

## Material and Methods

### Animals

Eight- to twelve-week-old male C57BL/6 J mice were obtained from Clea (Tokyo, Japan) and maintained at Tohoku University Graduate School of Medicine. All mice were handled and all experiments were performed in accordance with the ARVO Statement Guidelines for the Use of Animals in Ophthalmic and Vision Research and the Committee for Animal Experiments at the Tohoku University Graduate School of Medicine. The protocol number approved by our institute is 2017-229.

### Induction of axonal injury in mice

For anesthesia, a mixture of ketamine (180 mg/kg) and xylazine (90 mg/kg) was used intramuscularly and NC was performed to induce damage to the RGCs, as previously described^4,63^. In brief, the optic nerve was exposed and crushed approximately 2 mm posterior to the eyeball with forceps for 5 s. After surgery, an ointment containing levofloxacin (Santen Pharmaceutical Co., Ltd., Osaka, Japan) was applied and the animals were kept on a heat pad. In all experiments, only the right eye was used.

### Immunohistochemistry

Staining with anti-RBPMS antibodies was performed after the mouse retinas were fixed with 4% paraformaldehyde. Cryosections were then stained as previously described^64^. Briefly, the cryosections were incubated with rabbit anti-RBPMS (Abcam, #194213; dilution 1:200) and then incubated with goat anti-rabbit IgG Alexa Fluo 488. Nuclear staining was performed with Vectashield, including DAPI (Vector). Immunofluorescence images were captured through a microscope (Axiovert 200; Carl Zeiss, Berkochen, Germany) and immunofluorescence images of the entire retina were obtained and quantified with a fluorescence microscope (BZ-9000; Keyence, Osaka, Japan).

### Western blotting

The mouse retinas were homogenized, the extracted protein concentration was calculated, the retinal proteins were separated with SDS-PAGE, and the retinal proteins were transferred to a PVDF membrane, according to a method previously described^65^. The membrane was blocked with 1% skim milk in Tw-PBS for 1 h at room temperature and then incubated with rabbit anti-RBPMS antibody (Abcam; dilution 1:1000) overnight at 4 °C. After washing the membranes with Tw-PBS, they were incubated with HRP- conjugated donkey anti-rabbit IgG (Sigma; dilution 1:5000) at room temperature for 1 h. The immunoreactive signal was developed with ECL prime reagent (GE Healthcare, Piscataway, NJ) and was captured with ChemiDoc (Bio-Rad). The membranes were then reblotted with Restore Western Blot Stripping Buffer (Thermo Scientific) and incubated with mouse anti-beta-actin antibody (Sigma; dilution 1:5000) as an internal control.

### Reagents for LC-MS analysis

Methanol, chloroform and acetonitrile for LC-MS were purchased from Kanto Chemical (Tokyo, Japan). Ammonium formate (1 mol L-1) and formic acid for LC-MS were purchased from Wako Pure Chemical Industries (Osaka, Japan). Chemical standard phosphatidylcholine (PC) was purchased from Avanti Polar Lipids (Alabaster, AL). Chemical standards including spermine, carnitine and α-cyano-4-hydroxycinnamic acid (CHCA), were purchased from Sigma-Aldrich (Tokyo), and 9-Aminoacridine (9-AA) was purchased from Merck Schuchardt (Hohenbrunn, Germany). All chemical standards were obtained from common commercial sources.

### Sample preparation for LC-MS analysis

For the G-met analysis, 8 mouse retinas were used per condition for the controls and for NC day 2, and 4 mouse retinas were used per condition for NC day 4 and day 7. The obtained retinal samples were placed into sample tubes (2.0 mL). Two hundred µL of methanol containing 0.1% formic acid was added to the frozen sample. The mixture was homogenized using a lysis and homogenization system (Precellys) (5,000 × rpm, 15 s, 2 Zr bead). After homogenization in an ultrasonic bath for 10 min, the samples were then centrifuged at 16,400 g for 20 min at 4 °C, and the supernatant was passed through a 96-well plate for deproteinization (Sirocco, Waters Corp.) and then washed 3 times with 100 µL of methanol containing 0.1% formic acid. Thirty µL of each sample was collected from the 96-well plate and mixed in a 15-mL tube. Then, the mixture was transferred into a well as a study quality control (SQC). From SQC, a series of dilution quality controls (dQC) were prepared by dilution with 50% methanol containing 0.1% formic acid as follows: dilution at 2-fold (d2QC), 4-fold (d4QC), 8-fold (d8QC), and 16-fold (d16QC). Finally, the dilution plate was replicated; the original plate was used for UHPLC-QTOF/MS analysis, and the replicate plate was used for LC-FTMS analysis, respectively, with 4 µL and 3 µL sample sizes.

### Quality Control Sequences

The required frequency of SQC injections was determined with reference to previous reports^12^. The study-samples were injected in randomized run orders, and the SQC, which was mixed in all study-samples, was injected after every eight study-samples (2 h). In addition, 10 consecutive injections of SQC were made at the start of the chromatographic run to initialize the column. Finally, diluted (x times) QCs (dxQC) were injected three times at each concentration in the following order: d16QC, d8QC, d4QC, d2QC and SQC at the end of the sequence.

### UHPLC-QTOF/MS and LC-FTMS Methods

The UHPLC-QTOF/MS analysis was performed on an Acquity Ultra Performance LC I-class system, equipped with a binary solvent manager, a sample manager, and a column heater (Waters Corp.). This system interfaced with a Waters Synapt G2-Si QTOF MS with electrospray ionization (ESI) system, operated in both positive and negative ion modes. LC separation was performed using a C18 column (Acquity HSS T3; 150 mm × 2.1 mm i.d., 1.8 µm particle size; Waters) with a gradient elution of solvent A (water containing 0.01% formic acid) and solvent B (acetonitrile containing 0.01% formic acid) at 400 µL min^−1^. The data were collected using MassLynx, v4.1 software (Waters Corp., Manchester, UK).

The LC-FTMS system consisted of a NANOSPACE SI-II HPLC, equipped with a dual pump system, an auto sampler, and a column oven (Shiseido, Tokyo, Japan), and a Q Exactive Orbitrap MS (Thermo Fisher Scientific, San Jose, CA) equipped with a heated-ESI-II (HESI-II) source for positive and negative ion mode analysis. LC separation was performed using a HILIC column (ZIC^R○^-pHILIC; 100 mm × 2.1 mm i.d., 5 µm particle size; Sequant, Darmstadt, Germany) with a gradient elution of solvent A (10 mmol L^−1^ ammonium bicarbonate in water, pH 9.2) and solvent B (acetonitrile) at 300 µL min−1. The data were collected using Xcalibur v4.1 software (Thermo Fisher Scientific, San Jose, CA). Details of the UHPLC-QTOF/MS and LC-FTMS operating conditions can also be found in previous reports^12^.

### Data Processing

All data obtained from the four assays in the two systems, in both the positive and negative ion modes, were processed with Progenesis QI data analysis software (Nonlinear Dynamics, Newcastle, UK) for peak picking, alignment, and normalization, to produce peak intensities for *t*_R_ and m/z data pairs. The range of automatic peak picking for the C18 and HILIC assays was between 0.5 and 13.0 min and between 0.5 and 9.0 min, respectively; the ‘more 5’ mode was selected in setting the threshold for the sensitivity of picking. Then, the adduct ions of each “feature” (m/z, *t*_R_) were deconvoluted, and these features were identified from the human metabolome database (HMDB) and Lipidmaps. Features were selected based on their coefficient of variation (CV) with the SQC samples, which were injected after every 8 study samples; features with CV over 30% were eliminated. Features were also positively selected according to the inverse correlation of the dilution fold and the peak intensity to the dQC samples, as well as their CV with 3 injections of the same dQC samples. Then, the values of the compounds were imported to the Quantbolome (software) for log-median-regression. The normalization process has been described previously^12^. Finally, the values were normalized to the volume (in mg) of retinal material.

### Hierarchical Cluster Analysis

A heat map was generated for the list of z scores for each metabolite, which were selected with the Kruskal-Wallis test (*p* < 0.04) when the CV was under 30% in each group (Ctrl, NC day 2, NC day 4 and NC day 7), using gplots package in the R program (v. 3.2.0). A dendrogram of the metabolites was made with the complete linkage clustering method and correlation distance measuring with the amap package. The colored scale bar, running from blue to white and red, represents low, medium, and high intensity metabolites, respectively.

### Sample preparation for IMS analysis

Eight-µm sections of the eye were obtained from the mice with a cryostat (CM 3050 S; Leica Microsystems, Wetzlar, Germany) and set on indium-tin oxide slides (100 ohm/sq; Matsunami, Osaka, Japan). The slides were then put into 50-mL plastic tubes with silica gel and the matrix was applied. Regions of the tissue samples exposed to laser irradiation were identified by light microscopic observation. Then, 660 mg of CHCA or 9-AA was deposited on the slides, at a thickness of 1.4 μm in an iMLayer (Shimadzu, Kyoto, Japan), to analyze the positive ion mode and negative ion mode. The slide glass was set on a box (cm x cm x cm) with a filter paper, which was impregnated with 350 µL of water/methanol = 95/5 (v/v). Then, the sample was incubated at 85 °C for CHCA, or 40 °C for 9-AA for 3 min. The sample was then dried in a desiccator for 30 min. The samples were immediately analyzed with MALDI-IMS (iMScope, Shimadzu).

### MALDI-IMS analysis

MALDI-IMS analysis was performed with iMScope (Shimadzu). The mass spectra of the designated areas on a specimen photographed before matrix application were acquired in the positive and negative ion modes. Mass spectra were acquired under the following conditions: laser frequency and scanning mass ranged from *m/z* 130 to 280, *m/z* 500 to 750 and *m/z* 750 to 1,000 for positive, or *m/z* 750 to 1,000 for negative. Regions of the tissue samples exposed to laser irradiation were identified by light microscopic observation. The laser irradiation time, laser power, laser irradiation diameter, laser frequency, detection voltage, sample voltage and accumulated number of MALDI-IMS were 100 shots, 22 (pos) and 21 (neg), 10 μm, 1000 Hz, 2.1 kV, 3.5 (pos) and 3.0 (neg) and 1/pixel, respectively. A raster scan on the tissue surface was performed automatically. The number of pixels per scan was 57 × 53 (Ctrl) and 61 × 53 (NC day 2) for m/z 130 to 280, 68 × 53 (Ctrl) and 70 × 53 (NC day 2) for m/z 500 to 750, 55 × 52 (Ctrl) and 38 × 53 (NC day 2) for m/z 750 to 1,000 with the positive ion mode, and 69 × 48 (Ctrl) and 43 × 53 (NC day 2) with the negative ion mode. The spatial interval of data points was 10 μm, giving 3,121, 3,233, 3,604, 3,710, 2,860, 2,014, 3,312 and 2,279 data points in total for each section. The metabolites were identified with the MS/MS spectrum using the following chemical standards: spermine and PCs. The data collected through the microscopic system were digitally processed with imaging MS solution analysis software (Shimadzu). The mass spectrum signal intensity at each point in the IMS analysis in the selected region of the GCL was detected automatically after a region of interest (ROI) was set with MS solution imaging software. The signal intensity in the ROI was measured with a slight modification of a method reported in previous work^66^. In total, an 18-point area in the controls and a 26-point area for NC day 2 were used to measure L-acetylcarnitine, a 143-point area was used for the controls, and a 120-point area was used for NC day 2 to measure PC (38:7) in two mouse retinas.

### Statistical analysis

The statistical significance of the number of RBPMS-positive cells was determined with Dunnett’s multiple comparison test. P values < 0.05, compared to uninjured controls, were considered to be statistically significant. The intensities of the identified features were imported to the SIMCA 13.0 software (Umetrcxs, Umea, Sweden) for the multivariate analysis, and their relative quantities were evaluated with a PCA and an OPLS-DA. P-values were calculated with the Student’s t-test or Wilcoxon rank sum test. For IMS analysis, *P* values were calculated with the Student’s *t*-test.

## Electronic supplementary material

Supplementary information
