# Supplementary material for: Metabolomic changes in the mouse retina after optic nerve injury
Source: Sci Rep. 2018 Aug 9;8:11930. doi: 10.1038/s41598-018-30464-z (PMC6085332; doi:10.1038/s41598-018-30464-z)
Supplement: Supplementary file 1 — Supplementary information [file 41598_2018_30464_MOESM1_ESM.pdf]

<Title>

Metabolomic changes in the mouse retina after optic nerve injury

<Authors>

Kota Sato[1,2,a], Daisuke Saigusa[3,4,5,a], Ritsumi Saito[3,4], Amane Fujioka[1], Yurika Nakagawa[1], Koji M Nishiguchi[6], Taiki Kokubun[1], Ikuko N. Motoike[3,7], Kazuichi Maruyama[8], Kazuko Omodaka[1,2], Yukihiro Shiga[1], Akira Uruno[4], Seizo Koshiba[3,4], Masayuki Yamamoto[3], Toru Nakazawa[1,2,6,9,*]

1 Department of Ophthalmology, Tohoku University Graduate School of Medicine, Miyagi, Japan
2 Department of Ophthalmic imaging and information analytics, Tohoku University Graduate School of Medicine, Miyagi, Japan
3 Department of Integrative Genomics, Tohoku Medical Megabank Organization, Tohoku University, Sendai, Miyagi, Japan,
4 Medical Biochemistry, Tohoku University School of Medicine, Sendai, Miyagi, Japan
5 CREST, Japan Agency for Medical Research and Development (AMED), Chiyoda, Tokyo, Japan
6 Department of Advanced Ophthalmic Medicine, Tohoku University Graduate School of Medicine, Miyagi, Japan
7 Department of Systems Bioinformatics, Graduate School of Information Sciences, Tohoku University, Sendai, Miyagi, Japan
8 Department of Innovative Visual Science, Graduate School of Medicine, Osaka University, Japan
9 Department of Retinal Disease Control, Tohoku University Graduate School of Medicine, Miyagi, Japan

a K. Sato and D. Saigusa contributed equally to this work.

Correspondence to: Toru Nakazawa, Department of Ophthalmology, Tohoku University Graduate School of Medicine, 1-1, Seiryo, Aoba, Sendai, Miyagi 980-8574, Japan; Phobe; +81 22 717 7294; Fax: +81 22 717 7298; E-mail: ntoru@oph.med.tohoku.ac.jp (T. Nakazawa).

Supplementary Figure 1

Distribution of representative metabolites (listed in Fig. 2), as identified with G-Met assays. The level of the following metabolites was not significant in the retinas of the NC day 2 group, as measured with imaging mass spectrometry (IMS) assays: (a) m/z 136.06, m/z 258.11, m/z 268.10 for adenine, glycerophosphocholine, adenosine; (b) m/z 520.33, LPC 18:2; and (c) m/z 723.49, phosphatidylglycerol 32:0. A, B, and C are microscopic images showing hematoxylin-eosin staining of cryosections of the eyes (x 2.5), with the retinal area around the optic disk at higher magnification (x 20). These images were obtained after IMS. Relative intensities are shown by the colored scale bar on the left side of the IMS data.

Supplementary Table 1

List of features that differed significantly in the OPLS-DA data between the control and NC groups, as measured with an LC-MS assay.

Supplementary Figure 1

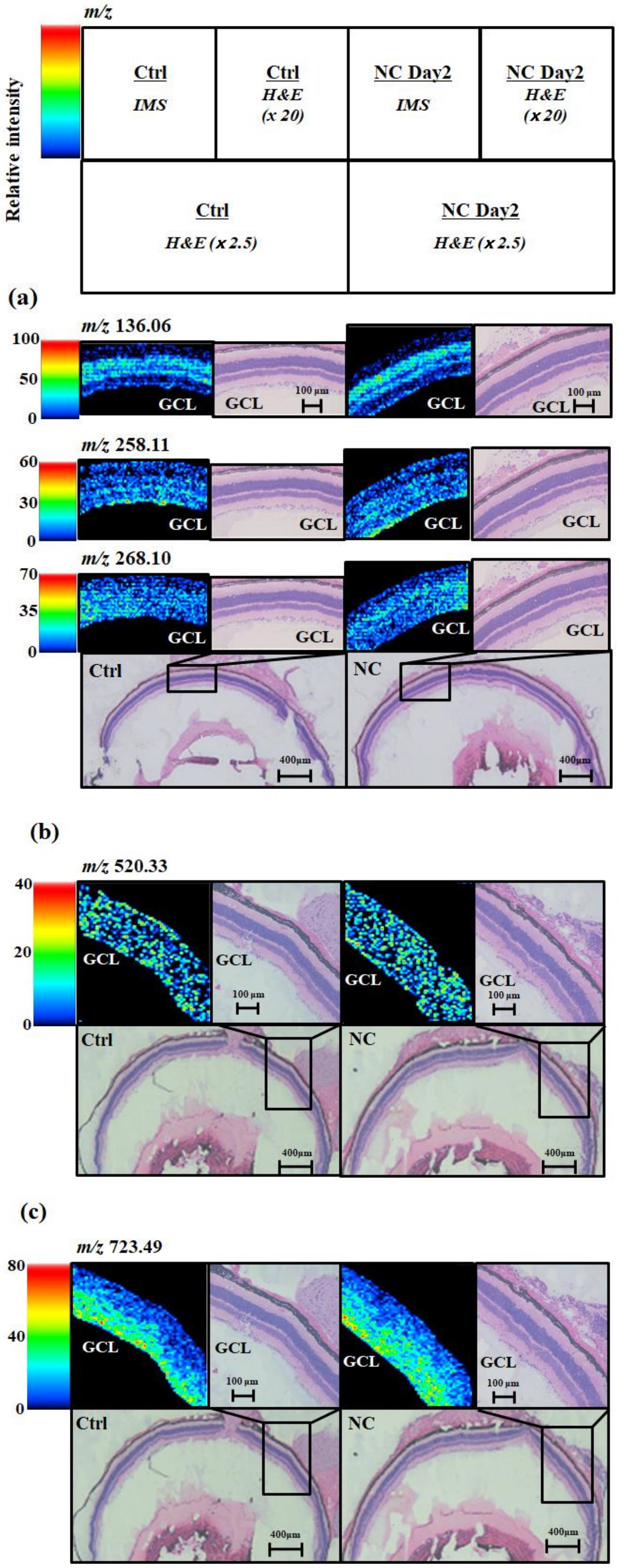

Supplementary Table 1

Analytical Mode: C18 column-positive ion mode							
High in NC Day2				Low in NC Day2			
Retention Time	*m/z*	p[1]	p(corr)[1]	Retention Time	*m/z*	p[1]	p(corr)[1]
0.717	480.8342	0.03371	0.61717	0.696	378.9002	-0.06626	-0.64979
0.717	666.8230	0.04165	0.72154	0.703	446.8876	-0.08935	-0.62980
0.717	986.6923	0.06366	0.68499	0.703	396.8731	-0.07664	-0.68492
0.724	646.7582	0.07378	0.74340	0.703	868.7158	-0.06927	-0.68793
0.724	866.6946	0.04192	0.67380	0.710	106.9509	-0.07121	-0.61226
0.724	494.8095	0.09219	0.70569	0.710	598.8363	-0.06107	-0.61275
0.724	578.7709	0.06090	0.71697	0.710	174.9383	-0.03342	-0.63317
0.724	798.7089	0.04740	0.69098	0.710	1054.6769	-0.07489	-0.66828
0.724	496.8081	0.04065	0.66009	0.710	310.9129	-0.07520	-0.67020
0.724	530.8484	0.04103	0.68294	0.717	90.9772	-0.06874	-0.67463
0.731	614.8100	0.02462	0.60072	0.724	730.7205	-0.06074	-0.62958
0.731	882.6704	0.03893	0.75690	0.724	122.9249	-0.03822	-0.64352
0.731	580.7697	0.04976	0.71344	0.724	342.8609	-0.07914	-0.65199
0.731	512.7820	0.03609	0.62666	0.724	276.8717	-0.06184	-0.66236
0.731	950.6571	0.04818	0.74060	0.724	274.8734	-0.06459	-0.68454
0.731	662.7330	0.04536	0.74537	0.724	834.7453	-0.06151	-0.68738
0.731	208.8843	0.04941	0.62138	0.731	290.8472	-0.06698	-0.63342
0.731	632.7839	0.07629	0.73759	0.817	539.0218	-0.03976	-0.67022
0.738	526.7576	0.06250	0.75967	0.824	876.1099	-0.06946	-0.62609
0.746	104.1074	0.04227	0.66858	0.824	663.5968	-0.03207	-0.72973
0.753	175.1194	0.03013	0.64855	0.873	160.0959	-0.14655	-0.55199
0.753	399.1446	0.03502	0.61828	6.870	566.9315	-0.12519	-0.59673
0.760	170.0926	0.02743	0.61568	6.933	589.8139	-0.13850	-0.57931
0.788	104.0711	0.04098	0.69852	6.969	170.1546	-0.19690	-0.65919
0.810	233.1499	0.03003	0.66063	7.083	334.7624	-0.21148	-0.66092
0.817	637.5726	0.03792	0.75834	7.431	284.0941	-0.20697	-0.64611
0.824	1039.0814	0.02749	0.65848	7.474	283.1531	-0.07227	-0.64722
0.853	515.2130	0.26726	0.84119	7.502	351.1795	-0.13165	-0.62918
0.873	159.0768	0.02386	0.66496	8.989	161.0968	-0.04054	-0.67983
0.873	205.0684	0.02277	0.67736	8.996	267.1945	-0.09448	-0.74995
0.888	235.1656	0.03463	0.64504	9.131	290.1606	-0.10236	-0.60661
0.931	315.1433	0.06445	0.77340	9.160	214.1623	-0.11019	-0.62140
0.931	90.0554	0.02683	0.70558	9.160	329.0054	-0.13379	-0.64264
0.945	177.9927	0.12128	0.75894	9.195	370.3319	-0.13557	-0.62996
0.952	489.0456	0.14579	0.75986	9.223	338.2672	-0.12590	-0.60611
0.966	272.9255	0.02927	0.71583	9.273	235.1328	-0.11067	-0.60457
0.981	398.9771	0.05943	0.78474	9.280	413.4106	-0.09191	-0.63424
1.044	457.9582	0.04211	0.67832	9.330	399.1232	-0.09867	-0.59157
1.087	251.9499	0.16478	0.83749	9.330	289.0440	-0.09935	-0.59365
10.198	297.2446	0.09067	0.71869	11.229	496.4210	-0.09840	-0.68318
				11.379	333.2767	-0.11020	-0.59742
